# Incestuous Sisters: Mate Preference for Brothers over Unrelated Males in *Drosophila melanogaster*

**DOI:** 10.1371/journal.pone.0051293

**Published:** 2012-12-10

**Authors:** Adeline Loyau, Jérémie H. Cornuau, Jean Clobert, Étienne Danchin

**Affiliations:** 1 CNRS, Station d’Ecologie Expérimentale du CNRS à Moulis, USR 2936, Saint Girons, France; 2 CNRS, Université Paul Sabatier, ENFA, Laboratoire d'Evolution et Diversité Biologique (EDB), UMR 5174, Toulouse, France; 3 Université de Toulouse, UMR 5174, Toulouse, France; 4 Department of Conservation Biology, Helmholtz Centre for Environmental Research – UFZ, Leipzig, Germany; Aarhus University, Denmark

## Abstract

The literature is full of examples of inbreeding avoidance, while recent mathematical models predict that inbreeding tolerance or even inbreeding preference should be expected under several realistic conditions like e.g. polygyny. We investigated male and female mate preferences with respect to relatedness in the fruit fly *D. melanogaster*. Experiments offered the choice between a first order relative (full-sibling or parent) and an unrelated individual with the same age and mating history. We found that females significantly preferred mating with their brothers, thus supporting inbreeding preference. Moreover, females did not avoid mating with their fathers, and males did not avoid mating with their sisters, thus supporting inbreeding tolerance. Our experiments therefore add empirical evidence for inbreeding preference, which strengthens the prediction that inbreeding tolerance and preference can evolve under specific circumstances through the positive effects on inclusive fitness.

## Introduction

Until recently, it has been the norm to assume that inbreeding avoidance should be the rule of thumb in mate choice under most circumstances because mating with relatives often causes a reduction in the fitness of inbred offspring. This reduction of fitness, which is called inbreeding depression, is due to either a loss of heterozygosity or an increased expression of recessive deleterious alleles [1]. The fitness costs of inbreeding are well-documented in animals and plants, and notably lead to a decrease in offspring birth weight, survival, fecundity, low resistance to pathogens, reduction in behavioural and morphological sexual traits or an increased susceptibility to predation or environmental stress (reviewed by [2], [3]). These impacts of inbreeding depression are largely context-dependent. They are greater in stressful than benign environments [4]. They also depend on the genetic diversity and inbreeding history of the study population, which can induce strong differences between species or populations [5]–[7]. For example, outbred populations of *Drosophila melanogaster* that experienced ancestral inbreeding were able to purge the deleterious alleles thanks to natural selection, which reduced the level of inbreeding depression [6]–[7].

Dispersal and kin recognition are therefore assumed to have evolved to avoid inbred mating [8], [9], and evidence of inbreeding avoidance via mate choice or preference for genetically dissimilar mate is plentiful (e.g. [9]–[24]). However, inbreeding might be as disadvantageous as outbreeding (i.e. mating with a genetically distant individual), due to the break down of locally adapted or co-adapted gene complexes [25], [11]. Therefore it has been suggested that individuals should prefer mating with individuals of intermediate relatedness to balance costs of both inbreeding and outbreeding [26]–[29].

Recently, Kokko & Ots [17] and Puurtinen [30] shed a new light on inbreeding avoidance by revisiting Hamilton’s inclusive fitness theory [31], initially developed to explain social evolution and eusociality. Also known as kin selection theory, this theory postulates that the inclusive fitness of an organism is equal to the sum of the increments in individual fitness of all relatives (including self) weighted by their relatedness to the organism [31]. Thus a female mating with a related male will gain direct fitness benefits from this mating (through her offspring), and indirect fitness by increasing the mating success of her relative mate. One condition is that such mating does not reduce mating opportunities for the male. As a consequence, inclusive benefits of mating with a relative are positively linked to relatedness between mates. These benefits are also higher in species in which both males and females mate multiply, as both genders will gain extra inclusive fitness from inbred mating. Using mathematical models, Kokko & Ots [17] and Puurtinen [30] showed that inbreeding avoidance is highly context-dependent. Inbreeding tolerance or even preference for related mates are likely to evolve in specific conditions, even if inbreeding depression imposes substantial costs, and thus should be more prevalent than previously appreciated. For example, it was predicted that sequential mate choice should favour inbreeding tolerance compared to simultaneous mate choice when mate availability is low (at least for one of the sexes), and that the sex that provides higher parental investment (usually males) should have higher inbreeding tolerance than the other sex [17]. Generally, the species expected to show the highest inbreeding tolerance are those in which mate availability is low and both males and females are able to breed multiple times [17].

If advantages of inbreeding tolerance can outweigh its costs under some circumstances, why is empirical evidence of inbreeding tolerance so limited? Kokko & Ots [17] suggested, among other explanations, that evidence would exist if we looked for it. Interestingly, since then, the list of examples of inbreeding tolerance is growing (e.g. [2], [32]–[41]). However, experimental evidence of mating preferences for close relatives is still scarce (e.g. bird [26]; cestode [42]; insects [3]; fish [43]). Experimental tests represent the best way to explore inbreeding avoidance and tolerance. Indeed, investigations in wild populations may not always provide clear-cut results. For example, genetic data revealed non-random mating with respect to relatedness in a natural population of *D. melanogaster*, as males appeared more likely to be mating with a related female, but this finding may result from an overall elevated relatedness between all sampled individuals (not just between mating pairs) ([44] see also [45] for a similar issue), and it is still not clear whether this finding results from female or male mate choice. Robinson and colleagues [44] therefore concluded that an experimental approach exploring mate preferences with respect to relatedness is needed in this species.

Here, we investigated the strength and the direction of mate preference for relatedness in both male and female *D. melanogaster*. *D. melanogaster* is an ideal species to examine inbreeding avoidance and tolerance. Both males and females mate multiply [46]. Experimental isofemale lines are generated by mating first-order relatives (full-siblings), showing that a high relatedness does not preclude reproduction and maintenance of highly inbred populations in laboratory conditions. Moreover, a field study revealed potential male preference for related mates, with lower number of offspring produced as an associated cost of inbreeding [44]. Additional costs of inbreeding may be a reduced sperm competitive ability [47] (but see [48]–[49] for contrasting results). Finally, short generation time, capacity to control genetic relatedness and possibility to observe mate choice in the laboratory make *D. melanogaster* an ideal species to examine the effects of genetic relatedness on mate choice.

We performed laboratory mate choice experiments in which individuals were given simultaneous access to two individuals of the opposite sex, one related (full-sibling or parent) and one unrelated (with similar age and mating experience). We therefore tested four types of mate choice: 1) female choice between a brother and a non-brother male, 2) female choice between the father and a non-father male, 3) male choice between a sister and a non-sister female and 4) male choice between the mother and a non-mother female.

## Materials and Methods

### Culture Stocks and Generation of Experimental Males and Females

We used *D. melanogaster* of the wild type strain obtained from a large outbred laboratory population with overlapping generations. This population was derived from 2000 flies caught in Chavroches (France) in 2006 and was kindly provided by F. Méry in 2008. The flies were maintained at 20°C on a natural light:dark cycle, at low density and in standard 8 ml vials containing cornmeal-agar-yeast medium. All the flies were manipulated by gentle aspiration and were not anaesthetized.

To create 115 families from the stocks, we obtained virgin flies by separating males and females at emergence. Three days later, we randomly placed one female and one male into a 1 ml vial. The vials where no copulation occurred within 30 minutes were discarded. After copulation, each male (the father) and each female (the mother) were kept separately in an individual 8 ml vial containing food. Females were transferred on new vials containing food every four days. Resulting male and female offspring were sexed at emergence and placed separately in vials containing food. They were then used to test mate preferences 3 or 4 days later.

### Mate Choice Test

We aimed to investigate four types of mate choice: 1) female choice between a brother and a non-brother male, 2) female choice between the father and a non-father male, 3) male choice between a sister and a non-sister female and 4) male choice between the mother and a non-mother female. Choices for parent vs. unrelated individual were tested as early as possible, i.e. with offspring resulting from the first emergences to reduce parental age at experiment. This resulted in mothers and fathers being respectively 21±1 and 26±1 days old.

Tests were performed daily from 09:30 to 18:30. We used an experimental set up consisting of three transparent plastic vials (3.3 cm long and 1 cm diameter) separated by two removable microscope cover glasses, providing three compartments (as in [50]). Vials were cleaned with absolute ethanol and new glass partitions were used for each replicate. We placed the choosing individual (male or female) in the central compartment and one individual of the opposite sex in each peripheral compartment. We then removed the glass partitions and observed free interactions.

The focal individual had the choice between one related individual (either sibling or parent), and one unrelated individual of similar age and mating experience. Pairs of related and unrelated individuals were randomly chosen among every possible pair of families. All individuals involved in the mate choice test were used only once, but we sometimes tested mate choice of several male offspring and several female offspring of a given family. When this occurred we avoided full pseudo-replication by choosing the unrelated flies in another family.

To distinguish between same-sex individuals, flies were marked with either a green or a pink colour powder as in [50]–[51], by introducing them into a vial containing traces of powder. They were then transferred into a clean vial containing food where they stayed for at least 45 min before experiment. This delay allowed them to clean so that only few colour spots dusted their thorax and back at the onset of the experiment. Coloration had no impact on mate choice (GLM, female choice: Khi^2^_1,220_ = 0.00, p = 1.00; male choice: Khi^2^_1,53_ = 1.80, p = 0.1794). Related and unrelated individuals were alternately placed in the left or right peripheral compartments, and were alternately green or pink.

**Figure 1:**
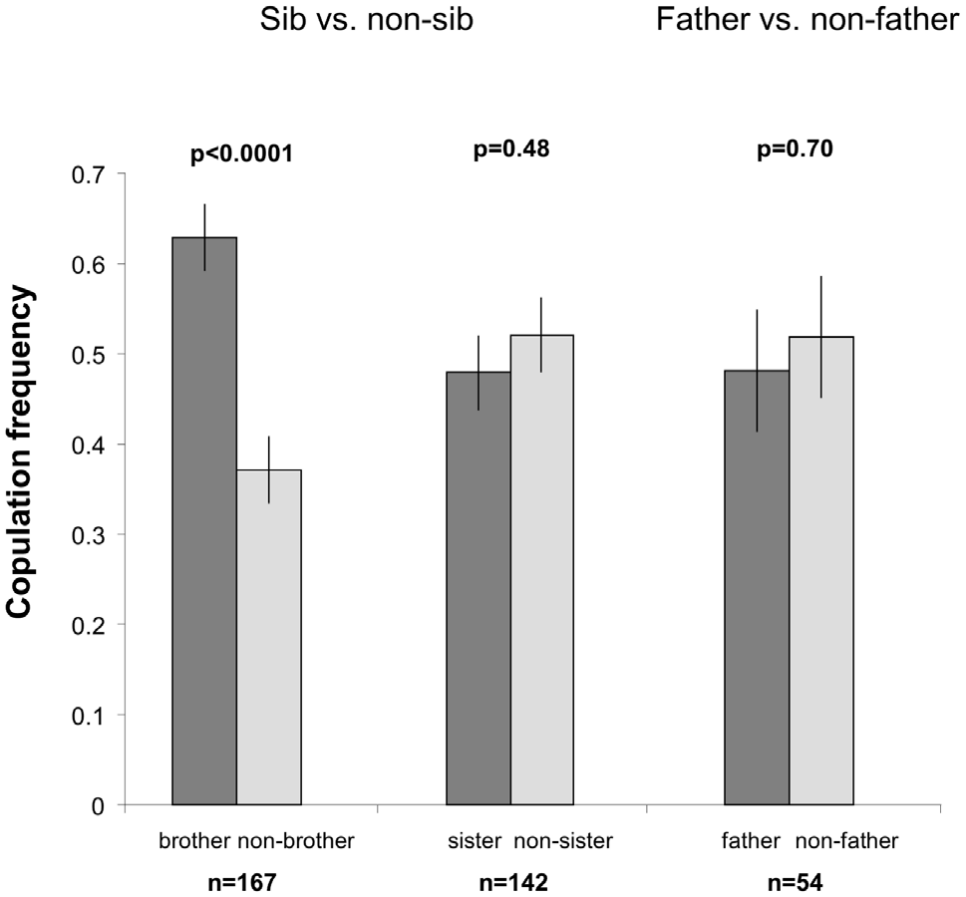
Copulation frequency for brother vs. non-brother, sister vs. non-sister and father vs. non-father. No copulation occurred when we tested mother vs. non-mother.

**Figure 2:**
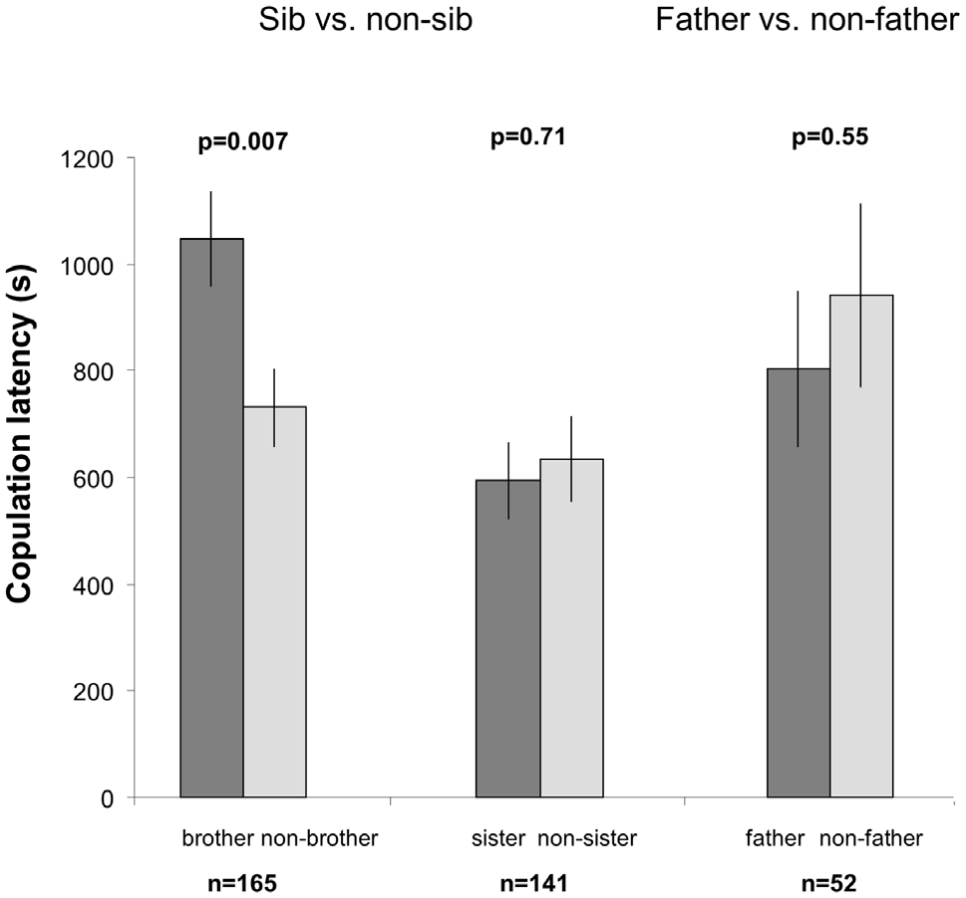
Latency to copulation for brother vs. non-brother, sister vs. non-sister and father vs. non-father. No copulation occurred when we tested mother vs. non-mother.

We measured mate preference by recording the identity (colour) of the chosen individual i.e. the individual who started to copulate with the focal individual. We also recorded the time from the removal of the partition glasses until copulation (latency to copulation). Male *Drosophila* intensively court virgin females, as a result females are expected to mate faster with more attractive males [52]. Because up to ten set ups were observed in parallel, the precise time of the beginning or end of the copulation could not be observed for some replicates, which accounts for discrepancies in the number of replicates between Fig. 1 and Fig. 2. Replicates in which no copulation occurred within 1 h were discarded from further analyses.

### Data Analyses

Statistical analyses were performed using SAS 9.1.3 (Cary, USA). We investigated mate choice for related versus unrelated individuals with generalized linear mixed models (GLMM, proc glimmix) with a binomial distribution of error terms (0/1 = unsuccessful/successful, link function: logit). The identity of the focal individual and the family identities were set as random factors to account for repeated sampling. Latency to copulation was investigated using generalized linear models (GLM, proc genmod) with a normal or Poisson distribution of effort terms (link functions: identity and log) and identity of the focal individual and family identities as repeated subjects. Represented values are means ± standard errors. Standard errors of frequencies were calculated as the following: s.e. = √[p(1-p)/n] where p and 1-p are the proportions and n the number of replicates.

## Results

We performed 167 replicates of brother vs. non-brother choice, 142 replicates of the daughter vs. non-daughter choice, and 54 replicates of the father vs. non-father choice during which copulation occurred. We also did 20 replicates of the mother vs. non-mother choice, in which all males were observed courting the 21±1 days old females, but no successful copulation occurred. This absence of copulation suggests that these females were still refractory at the time of experiment, i.e. 18 days after their first mating, and/or too aged to mate. This length for a refractory period is plausible in view of previous results [53]. Family identity was never significant (all ps>0.05).

When females were given the choice between a brother and an unrelated male, they mated significantly more often with their bothers (GLMM, F_1,166_ = 16.05, p<0.0001; Fig. 1). However, when they copulated with unrelated males, the latency to copulation was significantly lower compared to when they copulated with their brothers (GLM, d.f. = 1, Khi^2^ = 7.26, p = 0.0071; Fig. 2). When given the choice between a sister and an unrelated female, males tolerated mating with their sisters, as they did not show any preference between the two potential female mates (GLMM, F_1_,_141_ = 0.4797; Fig. 1). Male latency to copulation was comparable when mating to a sister and to an unrelated female (GLM, d.f. = 1, Khi^2^ = 0.14, p = 0.7128; Fig. 2). Similarly, females tolerated mating with their fathers as they did not show any preference between the two proposed males (GLMM, F_1,53_ = 0.15, p = 0.7019; Fig. 1) and latency to copulation did not differ between fathers and unrelated males (GLM, d.f. = 1, Khi^2^ = 0.36, p = 0.5458; Fig. 2).

## Discussion

We found evidence that *D. melanogaster* adult females preferred mating with their brothers over unrelated males with similar age and mating experience, which supports inbreeding preference in this species. Moreover, females tolerated mating with their fathers, and males tolerated mating with their sisters when they were given the choice between their father or their sister, and an unrelated individual, which supports inbreeding tolerance [17]. We also tried to investigate male mate choice between their mother and an unrelated female, but while males courted both females no copulation occurred (n = 20 replicates). Despite this relatively small number of replicates, the absence of copulation suggests that females were still refractory or aged and that such a mating is thus unlikely in nature implying that there may be no real selective pressure on such kind of choice.

Our experimental results confirms the non-random mating pattern with respect to genetic similarity found by Robinson et al. [44] in a natural population of *D. melanogaster*. It provides an additional experimental example of mate preference for inbreeding to the few ones that exist so far [3], [26], [42]–[43], and strengthens the prediction that inbreeding tolerance and mate preference for inbreeding are likely to evolve, as predicted through the positive effect on inclusive fitness [17], [30]. A female mating with her brother increases her inclusive fitness, while not restricting the mating opportunities of her brother because *D. melanogaster* males mate multiply [46]. On the contrary, copulation induces a refractory period during which the female cannot gain additional mating. This dissymmetry may explain why females, and not males, prefer mating with relatives in this species.

Despite the overall preference for mating with brothers, there was substantial variation among females when testing for the brother vs. unrelated choice. Interestingly, the latency to copulation was significantly lower for copulation with an unrelated male than with the brother. Latency to copulation is a good indicator of male attractiveness in this species [52], therefore our result suggests that females who preferred unrelated males over their brothers did so strategically and not for lack of a better option. One major explanation may be that, in *D. melanogaster*, female mate choice is known to be influenced by many morphological and behavioural male traits, including body size, comb size, courtship vigour, song frequency or pheromone production [54]–[56]. In our experiments, the unrelated males who were preferred over brothers may also have been of particular high quality with respect to these traits. In addition although the coefficient of relatedness for siblings is 0.5 on average, it can theoretically range from 0 to 1. Hence, females may simply copulate with unrelated males rather than with their brothers in those instances in which brothers and sisters were far too genetically similar.

If benefits in terms of inclusive fitness are sufficient to explain why *D. melanogaster* females prefer mating with their brothers, it is not clear why females did not also prefer mating with their fathers. This difference in mate preference is unexpected and somewhat surprising. One possibility is that, in our experiment, females could not discriminate their fathers from unrelated males. The probability that an individual meets its father or mother may be negligible in nature, thus there might be no selective pressure for such parent-offspring recognition. However, preference for relatives requires a mechanism of kin recognition. Given that females were able to recognise their brothers, we could reasonably suppose that they could also recognise their fathers. Several mechanisms have been proposed so far: family phenotype matching that implies that related individuals are used as references to perform a generalization against unrelated individuals, self-referent phenotype matching in which an individual uses its own phenotype as a reference against unrelated individuals, and recognition alleles that allow intrinsic recognition without learning (reviewed by [57]). In social and non-social insects, cuticular hydrocarbons (CHCs) are thought to play an important role in kin recognition [44]. A recent study in *D. melanogaster* unravelled that CHC profiles are strongly altered by aging, with the expression of long-chain CHCs increasing as individuals get older [58]. These changes of individual CHCs with age do not reflect a general deterioration in the capacity to produce individual CHCs because overall CHC levels increased [58]. However, they may affect a female’s ability to detect relatedness. Another possibility is that females did discriminate between fathers and unrelated males but based their mate choice on male characteristics other than relatedness, as already suggested above. Females are normally reluctant to mate with old males because courtship and mating abilities decline with age [58]–[60]. As males may exhibit inter-individual variation in the way they suffer from aging, females may use alternative male characteristics to do the best of a bad job, when having to choose between two old males.

One intriguing result of the mathematical model developed by Kokko & Ots [17] is that inbreeding tolerance and/or preference can be selected even when imposing substantial costs in terms of inbreeding depression, which is supported by empirical work [40], [43]. In our study, we did not measure the fitness outcome of the observed mating and thus we could not evaluate the direct costs of inbreeding. Whether observed mate preference may cause inbreeding depression in *D. melanogaster* is not clear yet. Robinson and colleagues [44], [61] found a negative relationship between the degree of genetic similarity within a pair and both egg-to-adult-viability and number offspring at emergence. Increased relatedness also decreased male ability to secure a mating when competing with non-inbred males [62]. However, in a population experiencing an intermediate level of inbreeding, as we may expect from our observations, a purging effect of deleterious alleles is likely to lower the impact of inbreeding, as experimentally found in *D. melanogaster* [63]. Our results were obtained from an experiment conducted with of a single population of *D. melanogaster*. As the impacts of inbreeding depression largely depend on environmental conditions and the genetic history of the population [4]–[7], further studies should investigate mate choice in regards to inbreeding in various environmental and genetic contexts. Populations that went through bottleneck(s) may have purged most deleterious alleles responsible for inbreeding depression and therefore be more prone to inbreeding tolerance or even preference, on the one hand. One the other hand, the loss of genetic diversity following bottlenecks may make kin recognition arduous, not allowing the evolution of inbreeding preferences.

To summarize, we performed experimental mate choice tests in the laboratory and found that *D. melanogaster* females prefer mating with their brothers over unrelated males. This result provides strong support for inbreeding preference, as benefits from inclusive fitness may outweigh the potential costs of inbreeding depression [17]. We also found that females tolerated mating with their fathers and that males tolerated mating with their sisters, which supports inbreeding tolerance.
